# Descriptor selection for predicting interfacial thermal resistance by machine learning methods

**DOI:** 10.1038/s41598-020-80795-z

**Published:** 2021-01-12

**Authors:** Xiaojuan Tian, Mingguang Chen

**Affiliations:** 1 Department of Chemical Engineering, China University of Petroleum, Beijing, 102249 China; 2 Physical Science and Engineering Division, King Abdullah University of Science and Technology (KAUST), Thuwal, 23955-6900 Saudi Arabia

**Keywords:** Materials science, Mathematics and computing, Nanoscience and technology

## Abstract

Interfacial thermal resistance (ITR) is a critical property for the performance of nanostructured devices where phonon mean free paths are larger than the characteristic length scales. The affordable, accurate and reliable prediction of ITR is essential for material selection in thermal management. In this work, the state-of-the-art machine learning methods were employed to realize this. Descriptor selection was conducted to build robust models and provide guidelines on determining the most important characteristics for targets. Firstly, decision tree (DT) was adopted to calculate the descriptor importances. And descriptor subsets with topX highest importances were chosen (topX-DT, X = 20, 15, 10, 5) to build models. To verify the transferability of the descriptors picked by decision tree, models based on kernel ridge regression, Gaussian process regression and K-nearest neighbors were also evaluated. Afterwards, univariate selection (UV) was utilized to sort descriptors. Finally, the top5 common descriptors selected by DT and UV were used to build concise models. The performance of these refined models is comparable to models using all descriptors, which indicates the high accuracy and reliability of these selection methods. Our strategy results in concise machine learning models for a fast prediction of ITR for thermal management applications.

## Introduction

Interfacial thermal resistance (ITR) plays an important role for thermal management of ultra-fast electronics and thermoelectric materials^1–6^. When heat is transferred through an interface, temperature discontinuity exits. The ratio of the temperature discontinuity to the heat flux through the interface is named as ITR. There is a miniaturization trend of electronic devices in recent decades. In terms of nanostructured devices, the ITR become a dominant factor for device performance because the phonon mean free paths are larger than the characteristic length scales under such circumstance^7^. Screening materials with desired ITR is significant for electronics fabrication. For example, materials system with low ITR helps to reduce the energy consumption of electronics, while high ITR materials system is required for excellent thermoelectrics. There are a great number of factors affecting ITR, such as the intrinsic properties of materials and their differences, roughness of surfaces, crystal impurities, binding energy and thickness of films etc^8,9^. Thus, the accurate prediction of ITR is a high-dimensional problem and difficult to solve with regular mathematic equations.

Traditional models predicting ITR include acoustic mismatch model (AMM) and diffuse mismatch model (DMM)^10^. AMM assumes that there is no scattering of photons at the interface, which works well only under ideal conditions at low temperature. The DMM model is built based on the assumption of complete elastic diffusing mismatch, which makes it not suitable for non-elastic circumstances. An improved model named scattering-mediated acoustic mismatch model (SMAMM) incorporates phonon scattering into the original AMM and realizes prediction of ITR in a wide temperature range^11^. Still, the prediction accuracy of SMAMM model is restricted by the Debye approximation and the reliability of experimental data used to fit the parameters^12^. Besides, Foygel’s model based on Monte Carlo simulations and percolation theory has been widely adopted to predict the thermal conductivity in carbon nanotube composites^13–17^. This model simplifies nanotubes as penetrable, rigid and straight cylinders and ignores the waviness and 3D entanglement of carbon nanotubes^18–20^. Molecular dynamics (MD) simulation is also applied to ITR prediction. Originally, MD simulation is used to analyze the physical movements of atoms and molecules. When it comes to a system of interacting particles, the system properties are predicted by numerically solving empirical or semi-empirical equations defined by classical Newton’s law of motion. The interactive forces between different particles are calculated following a potential function (eg. Lennard–Jones potential, tight-binding potential), which is an approximate function at a certain level of accuracy. The results of MD simulation are valid only when the input atomic interactions are consistent with the forces in real situations. In some simple cases, this assumption can be fulfilled by carefully select the potential functions. For example, Lennard–Jones potential can be selected for the non-bonded interaction between two particles^21^, while other potentials or methods such as embedded atom model^22^, environment dependent interatomic potential^23^, or tight-binding second moment approximation potentials^24^ can be adopted for many-body systems. However, it can be extremely hard to mimic forces between real atoms when quantum effects^25,26^, time^27^ and size limitations^28,29^ need to be taken into account in biologically important processes. Besides, MD simulation is computationally expensive and time-consuming, which limits its applications as screening tools for specific materials. Lately, machine learning methods have been applied to predict composite thermal conductivity, ITR between graphene and boron nitride, and thermoelectric conversion efficiency^30–38^. Specifically, Xu group^8^ applied machine learning algorithms as regression tree ensembles of LSBoost, support vector machines, and Gaussian regress processes to build ITR prediction models. Descriptors with a total amount of 35 including property descriptors, compound descriptors, and process descriptors were selected as input. All three models show better prediction accuracy than traditional AMM and DMM, which indicates the prospect of machine learning methods for predicting physical properties. However, it’s still very hard for researchers to consider all the 35 descriptors when designing thermal management systems with novel materials. In light of this, we focused on evaluating the 35 descriptors further by machine learning methods and screening minimum but most significant descriptors for ITR prediction.

For data set with modest size, descriptor selection is critical for reaching a robust machine learning model and provide insight on which characteristics are most important for the target^39,40^. In this work, descriptor selection was firstly conducted according to their importances calculated by decision tree (DT). The importances are the scores assigned to each input feature of a predictive model that indicates its relative contribution to the predicted results. And descriptors with topX(X = 20, 15, 10, 5) highest importances were selected (topX-DT). To verify the transferability of the selected subsets, kernel ridge regression (KRR), Gaussian process regression (GPR) and K-nearest neighbors (KNN) algorithms were used to build models besides DT. R^2^ and root-mean-squared-error (RMSE) of models built from descriptors subsets by all three algorithms were calculated. The metrics for model evaluation were acquired from shuffled and grouped cross-validation. Datasets were randomly split under shuffled cross-validation. Considering identical interface system may exist in both validation set and training set when shuffled, datasets were also grouped by substrate/interlayer/film system to exclude the potential interference on feature importance. It is shown that the performance of all algorithms are stable with descriptor size decreasing to top10-DT. DT has a relatively good performance even when the descriptor size reduces to top5-DT, while the performance of KRR, GPR and KNN is not satisfying. To obtain a more reliable feature subset, univariate selection (UV) was introduced. And the subset selected by UV is named as topX-UV. As a result, there are 15 common descriptors selected by both top20-DT and top20-UV (Top15-DTUV). Meantime, 5 common descriptors exist in both top10-DT and top10-UV (Top5-DTUV). It is confirmed that the model performance is more robust under descriptors selected both by DT and UV than that from descriptors only picked by DT. Besides, descriptors selected by DT and UV has a high overlap with the descriptors used for AMM and DMM and factors testified from previous experimental studies. Thus, the selected descriptors work well for building machine learning models and are valid on the physical point of view. Descriptor selection methods presented in this work are transferrable to predict other materials properties beyond ITR.

## Methods

### Dataset collection

Original dataset for this study was the experimental data collected from 85 published papers. Xu group organized them and introduced descriptors for predicting ITR by machine learning method^8,9^. Details of the developed descriptors and collected ITR were explained in the previous work^41^. And data could be found in the file named “training dataset for ITR prediction.xlsx” and downloaded directly from https://doi.org/10.5281/zenodo.3564173.

### Dataset preprocessing

Descriptors were scaled before feeding into models. According to distribution of descriptors, min–max scale and standard scale were applied. Min–max scale is to transform features by scaling each feature to a given range, e.g. between zero and one. For each descriptor, min–max scale is conducted by the following equation, where X.max and X.min are the maximum and minimum value of the descriptor.

$${\mathrm{X}}_{\mathrm{scaled}}=\frac{X-X.min}{X.max-X.min}$$

The descriptors transformed by min–max scaler include fthick, fmelt, fdensity, sdensity, fAC1x, fAC1y, fAC2x, fAC2y, fIPc, fIPa, smelt, sAC1x, sAC1y, sAC2x, sAC2y, sIPc, and sIPa.

Standard scale is to standardize features by removing the mean and scaling to unit variance. Centering and scaling happen independently on each feature by computing the relevant statistics on the samples in the training set. For each descriptor, standard scale is conducted by the following equation, where $\mu$ and s are the mean and standard deviation of the descriptor.

$${\mathrm{X}}_{\mathrm{scaled}}=\frac{X-\mu }{s}$$

The descriptors transformed by standard scaler are T, fmass, fEb, sEb, and smass.

### Algorithms and models

Decision trees (DT) are a non-parametric supervised learning method for classification and regression^42^. It creates a model to predict target by learning with a set of simple if–then-else rules. It is a white box model simple to understand, interpret, and visualize. A representative decision tree algorithm is classification and regression tree (CART), introduced by Leo Breiman^43^. CART is based on a binary recursive partitioning procedure. The objective of partitioning is to minimize dissimilarity in the terminal nodes for classification and mean-squared-error for regression. The dissimilarity is measured by the loss functions, typically Gini index or cross-entropy for classification trees^44,45^. For the regression trees applied in our work, each partitioning is made to maximize the reduction in root-mean-squared-error (RMSE)^46^. After all the partitioning has been done, a decision tree is obtained where each branch is a split in a predictor and each end node gives a prediction for the outcome variable. The feature importances in CART could be determined in one shot during training, which is computationally efficient compared with greedy search methods.

KRR is an algorithm combining Ridge regression (linear least squares with l2-norm regularization) with the “kernel trick”^47,48^. Actually, KRR is a special case of support vector regression. It takes advantage of integral operator kernel functions to map principal components in high-dimensional feature spaces to input space nonlinearly^49–51^. Radial basis function (RBF) was applied as kernel in our work.

GPR implements Gaussian processes for regression purposes. It can find a probabilistic distribution of new output given the training data and new input data^52–55^. Both KRR and GPR learn a target function by the “kernel trick”. However, KRR learns a linear function in the space induced by the respective kernel which corresponds to a non-linear function in the original space^49^. While, GPR uses the kernel to define the covariance of a prior distribution over the target functions and uses the observed training data to define a likelihood function. Here, we also applied radial basis function (RBF) as the kernel for the GPR^56^.

Besides descriptor importances from decision tree, feature selection was conducted by selecting the best descriptors based on the UV statistical tests for dimensionality reduction purpose. Here, F-test was adopted to estimate the degree of linear dependency between descriptor and target^57,58^. Briefly, F-test of equality of variances is a test for the null hypothesis that two normal populations have the same variance^59^. In this situation, F value is the ratio of descriptor variance over target variance. It has an F-distribution if the null hypothesis of equality of variances is true. If F value is either too large or too small, the null hypothesis will be rejected^60,61^. The built-in function f_regression of sklearn library computes the correlation between the descriptor and target, and converts it to an F value automatically. Then the F values are used for descriptor selection.

### Algorithm evaluation

Models built by different algorithms and descriptor subsets were evaluated by R^2^ and RMSE.

R^2^ computes the coefficient of determination. It is calculated by

$${R}^{2}=1-\frac{{\sum }_{i=1}^{n}{({y}_{i}-{u}_{i})}^{2}}{{\sum }_{i=1}^{n}{({y}_{i}-\stackrel{-}{u})}^{2}}.$$

And RMSE is calculated by

$$RMSE=\sqrt{\frac{{\sum }_{i=1}^{n}{({y}_{i}-{u}_{i})}^{2}}{n}}$$

where $n, {y}_{i},\boldsymbol{ }{u}_{i},$ and $\stackrel{-}{u}$ are number of data, experimental ITR, predicted ITR, and average experimental ITR values, respectively.

**Figure 1:**
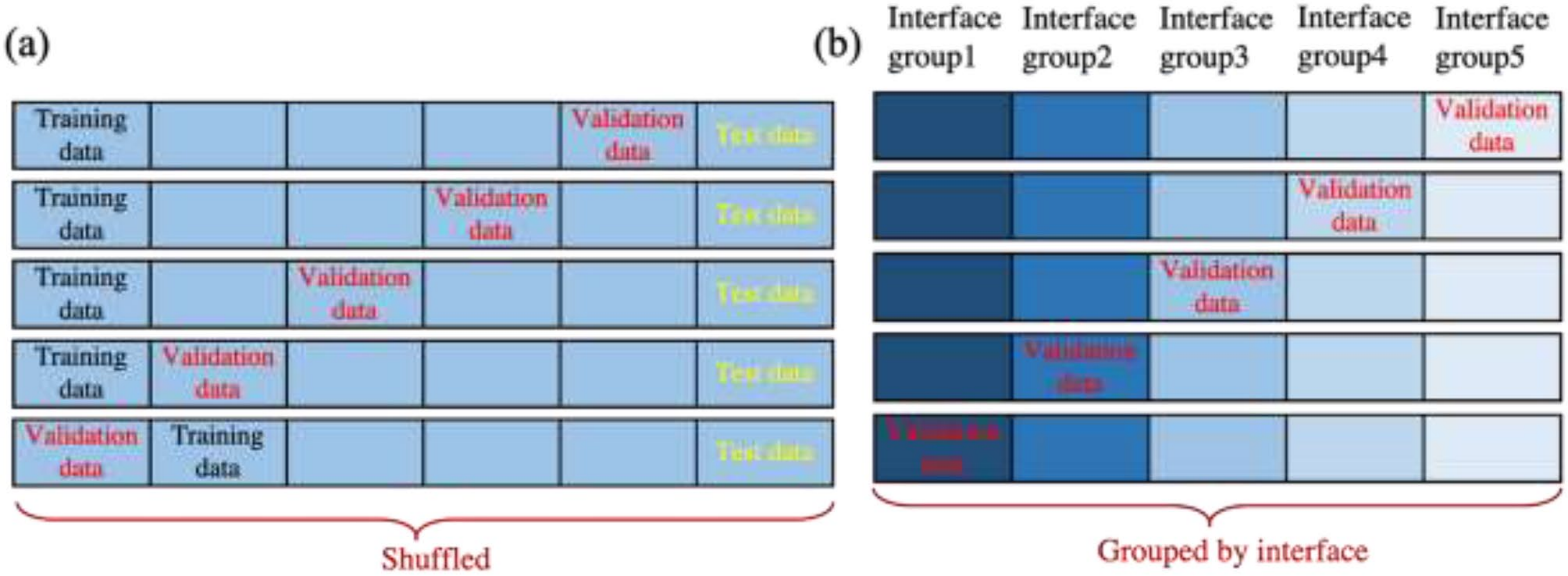
Schemes for (**a**) shuffled cross-validation and (**b**) grouped cross-validation.

The datasets were handed by shuffled cross-validation and grouped cross-validation for training and model evaluation, as seen in Fig. 1. In terms of the shuffled cross-validation, the original dataset was split into training and cross-validation set (80%) and holdout set (20%) randomly. Models were built by training set and optimal hyperparameters were picked through grid search with fivefold cross-validation. The holdout set was seen and used only once for model evaluation. Thus, the holdout set was named as test data. Under circumstances that the train and test set follow the same probability distribution, the holdout method can provide the most accurate metrics for unseen data, since the metrics obtained from validation set contain bias from hyperparameter optimization^62^. Besides shuffled cross-validation, the dataset was grouped by unique interfaces (film-interlayer-substrate). Every group contain ~ 20% dataset with some specific interfaces, which is different among these groups. Thus, no identical interface exists in more than one groups. In such case, data among these groups may follow different probability distribution. So the fivefold cross-validation was applied to evaluate model performance. R^2^ and RMSE were used as metrics in cross-validation.

Table S1 and S2 in supporting information summarize the grid search space and final hyperparameters picked for various models. Please refer to the github link (https://github.com/descriptor-selection-ITR/Descriptor-Selection-for-Predicting-Interfacial-Thermal-Resistance-by-Machine-Learning-Methods) for more details. Fivefold cross-validation was selected because our original dataset was less than 1000 samples. For such small datasets, fivefold cross-validation generally gives better results. Lower fold cross-validation can’t train the models well, while higher fold cross-validation allocates few data to test set, making testing results not representative. R^2^ and RMSE of predictions from test set were used to evaluate the performance of models.

All analysis were conducted in Scikit-lean package^63^. The StandardScaler and MinMaxScaler package was used for data preprocessing, DecisionTreeRegressor, KernelRidge, and GaussianProcessRegressor package for the three algorithms mentioned above, and SelectKBest and f_regression for univariate descriptor selection.

## Results and discussion

### Descriptors selected by decision tree

**Figure 2:**
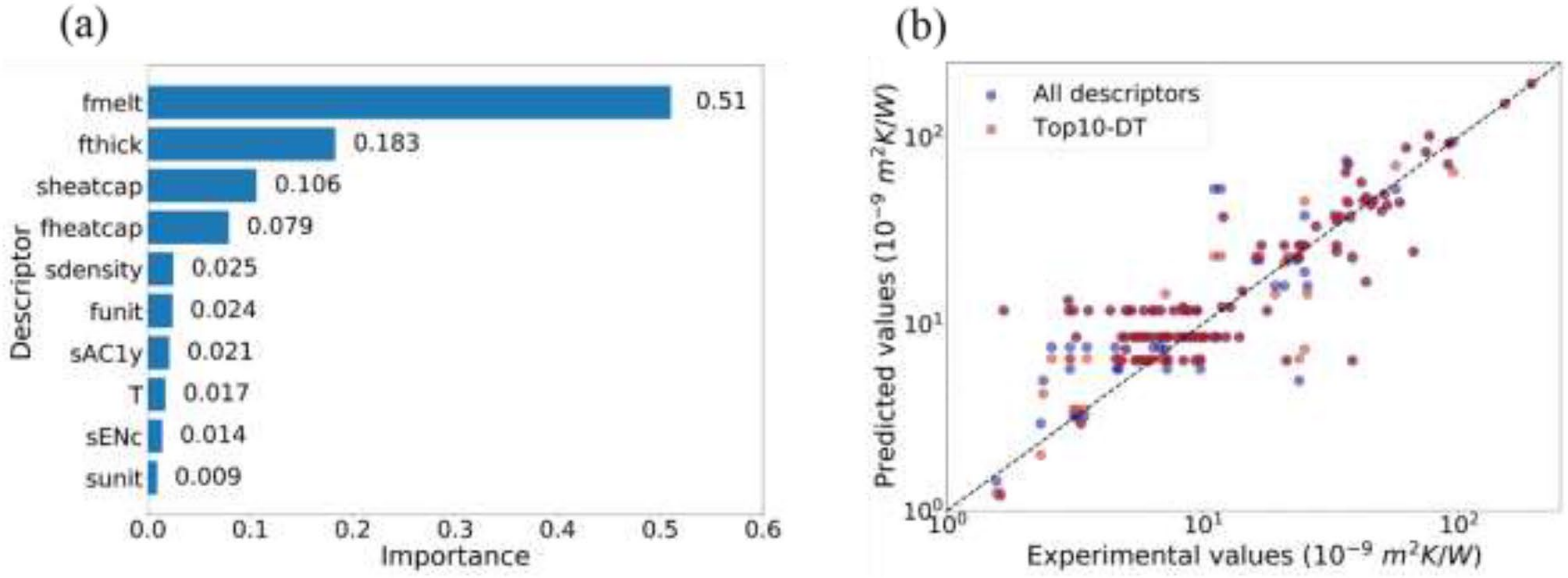
(**a**) Descriptors with the highest 10 importances (Top10 descriptors). (**b**) Correlation between the experimental values and values predicted by all descriptors (violet dots) and top10 descriptors (red dots).

Dataset was treated firstly by shuffled cross-validation with an ideal assumption that all data follow the same probabilistic distribution, as seen in Fig. 1a. Descriptor selection plays a critical role in building robust and computationally-cheap models. For dataset whose size is not large, descriptor selection is helpful to prevent overfitting and provide insight into which properties are most important for targets. Here, decision tree (DT) was applied to train an ITR prediction model and get the descriptor importances. Figure 2a shows the descriptors with the top10 highest importances (Top10-DT), which occupy a total importance of more than 98%. Among them, the film melting point has a high importance of 51%, and the top4 descriptors possess importance around 88%. Table 1 presents all the descriptors and their corresponding importances. Interestingly, only 20 out of 35 descriptors are selected by decision tree, indicating the existence of uninformative inputs. The descriptors for traditional AMM and DMM include temperature, density, speed of sound (longitudinal and transverse), and unit cell volume. It is worth noting that temperature, density and unit cell volume are all in the Top10-DT. Meanwhile, speed of sound (longitudinal and transverse) has a Pearson correlation coefficient as high as 0.71 with the melting point^9^, while melting point is the most important descriptor according to decision tree. Therefore, useful descriptors confirmed by AMM and DMM are all selected as important descriptors by decision tree successfully. As shown in Fig. 2a, heat capacity and film thickness also act as significant descriptors. The relationship between film thickness and ITR has been observed by experiments and simulations in previous studies^64,65^. The reason that heat capacity was selected is attributed to the relationship between heat capacity and density. Figure 2b shows the correlation between experimental values and predicted values of test data from DT. It is observed that there are same predicted values for multiple experimental data (horizontal series of data in Fig. 2b). This phenomena occurs since decision tree takes the mean of samples located at the same leaf node as prediction. Thus, the data assigned to the same leaf node has the same predicted value. It is indicated that the DT built from all descriptors and top10-DT have comparable performance.

**Table 1:** Descriptor importances from decision tree.

	Descriptor	Importance	Remarks
1	fmelt	$5.10\times {10}^{-1}$	interlayer: 1 (exist) or 0 (absent) T: temperature (K) fthick: film thickness (nm) The following columns are labeled for film and substrate by f and s in the front, respectively. (e.g. fheatcap and sheatcap) heatcap: specific heat capacity (J/gK) melt: melting point (K) density (g/cm^3) unit: Volume per formula unit (10^-29 m^3/f.u.) R1: atomic ratio of the first element R2: atomic ratio of the second element AC: AC represents atomic coordinates defined from the periodic table. The group as the x coordinate and the period as the y coordinate as (ACix, ACiy), where i represents the order of the elements of the compound ENc: electronegativity for cation ENa: electronegativity for anoin IPc: ionic potential for cation IPa: ionic potential for anion Eb: binding energy (eV/f.u.) mass (u)
2	fthick	$1.83\times {10}^{-1}$	
3	sheatcap	$1.06\times {10}^{-1}$	
4	fheatcap	$7.94\times {10}^{-2}$	
5	sdensity	$2.49\times {10}^{-2}$	
6	funit	$2.41\times {10}^{-2}$	
7	sAC1y	$2.14\times {10}^{-2}$	
8	T	$1.66\times {10}^{-2}$	
9	sENc	$1.37\times {10}^{-2}$	
10	sunit	$8.93\times {10}^{-3}$	
11	interlayer	$4.50\times {10}^{-3}$	
12	fAC1x	$3.07\times {10}^{-3}$	
13	fEb	$3.02\times {10}^{-3}$	
14	sAC2x	$5.73\times {10}^{-4}$	
15	fIPa	$5.34\times {10}^{-4}$	
16	sIPc	$4.48\times {10}^{-4}$	
17	fENc	$2.33\times {10}^{-4}$	
18	sR1	$3.47\times {10}^{-5}$	
19	smelt	$2.92\times {10}^{-5}$	
20	fdensity	$1.65\times {10}^{-7}$	
21	fmass	0	
22	sEb	0	
23	smass	0	
24	fAC1y	0	
25	fAC2x	0	
26	fAC2y	0	
27	fIPc	0	
28	sAC1x	0	
29	sAC2y	0	
30	sIPa	0	
31	fR1	0	
32	fR2	0	
33	fENa	0	
34	sR2	0	
35	sENa	0	
Total		1	

**Figure 3:**
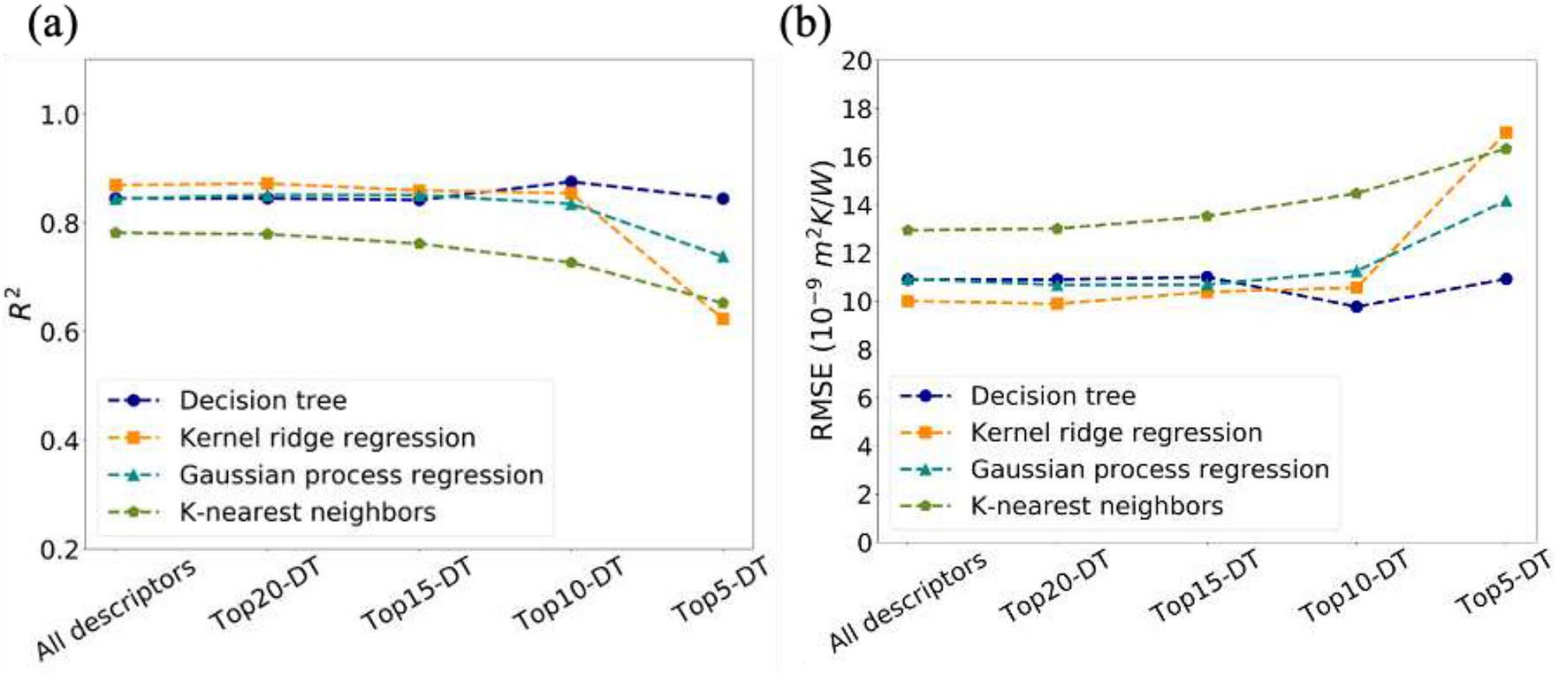
R^2^ (**a**) and RMSE (**b**) of test data (holdout set) predicted by models built by different descriptors subsets under shuffled cross-validation.

To verify the transferability of the descriptors selected by DT, kernel ridge regression (KRR), Gaussian process regression (GPR) and K-nearest neighbors (KNN) models were also built under different descriptor subsets. These subsets were named as topx-DT, presenting the descriptors with topx highest importances from DT, as shown in Table 1. Here, top20-DT, top15-DT, top10-DT, and top5-DT were applied as inputs together with all descriptors. Shuffled cross-validation was applied here. R^2^ and RMSE of the test data (holdout set) served as the metrics for model evaluation. It is believed that the performance on holdout set is the most close to that of unseen data, since hyperparameter optimization may result in overfitting to validation set. Commonly, a higher R^2^ and lower RMSE indicate a better performance. The R^2^ and RMSE for both training set and test set could be found in Table S3 in supporting information. As seen in Fig. 3, DT with all descriptors shows a R^2^ of 0.85 and a RMSE of 11, which is comparable to the previous results^8^. Notably, the performance of DT doesn’t degrade with the reduction of descriptors. In terms of KRR and GPR, the performance is as good as DT until top10-DT. When the descriptors size decreases further to 5, the performance of KRR and GPR degrades sharply. The performance of KNN model is not as good as the others. Overall, the top10-DT have a total importance of more than 98%, which include the properties used for AMM and DMM. These 10 descriptors have a good transferability from DT to other machine learning models, such as KRR and GPR.

### Descriptors selected by univariate testing

**Figure 4:**
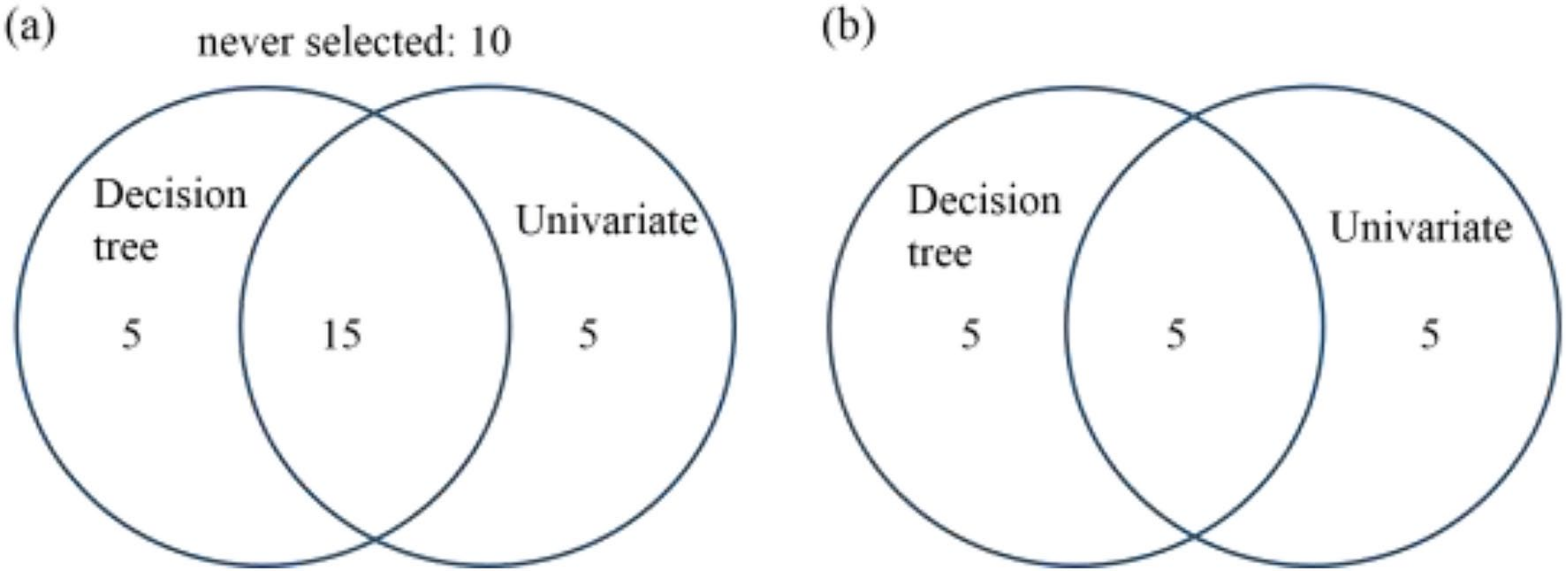
Venn diagram showing the amount of common descriptors picked for both decision tree and univariate testing.

To cross validate the descriptors selected by decision tree, univariate selection (UV) was applied. The UV is a totally different algorithms compared with decision tree selection. It filters descriptors based on statistical test. In this work, F-test estimating the degree of linear dependency between descriptor and targets was used. 10 and 20 out of 35 total descriptors were selected by univariate testing, as shown in Table S4 in supporting information. Figure 4 is the Venn diagram showing the amount of common descriptors for top 20 and top 10 descriptors selected by DT and UV. Obviously, there are 15 common descriptors out from 20 picked by both DT and UV. 10 descriptors are never selected. For the top10-DT and top10-UV, there are 5 common. The details of the 15 and 5 common descriptors are shown in Table 2. The 5 common descriptors for top10-DT and top10-UV include the melting point, heat capacity, unit and electronegativity.

**Table 2:** Common descriptors selected by decision tree and univariate testing.

	Descriptors
Top15-DTUV	fmelt, fthick, funit, fheatcap, fdensity, fEb, fENc, fAC1x, T, smelt, sunit, sheatcap, sAC1y, sENc, sIPc
Top5-DTUV	fmelt, fheatcap, funit, sheatcap, sENc

**Figure 5:**
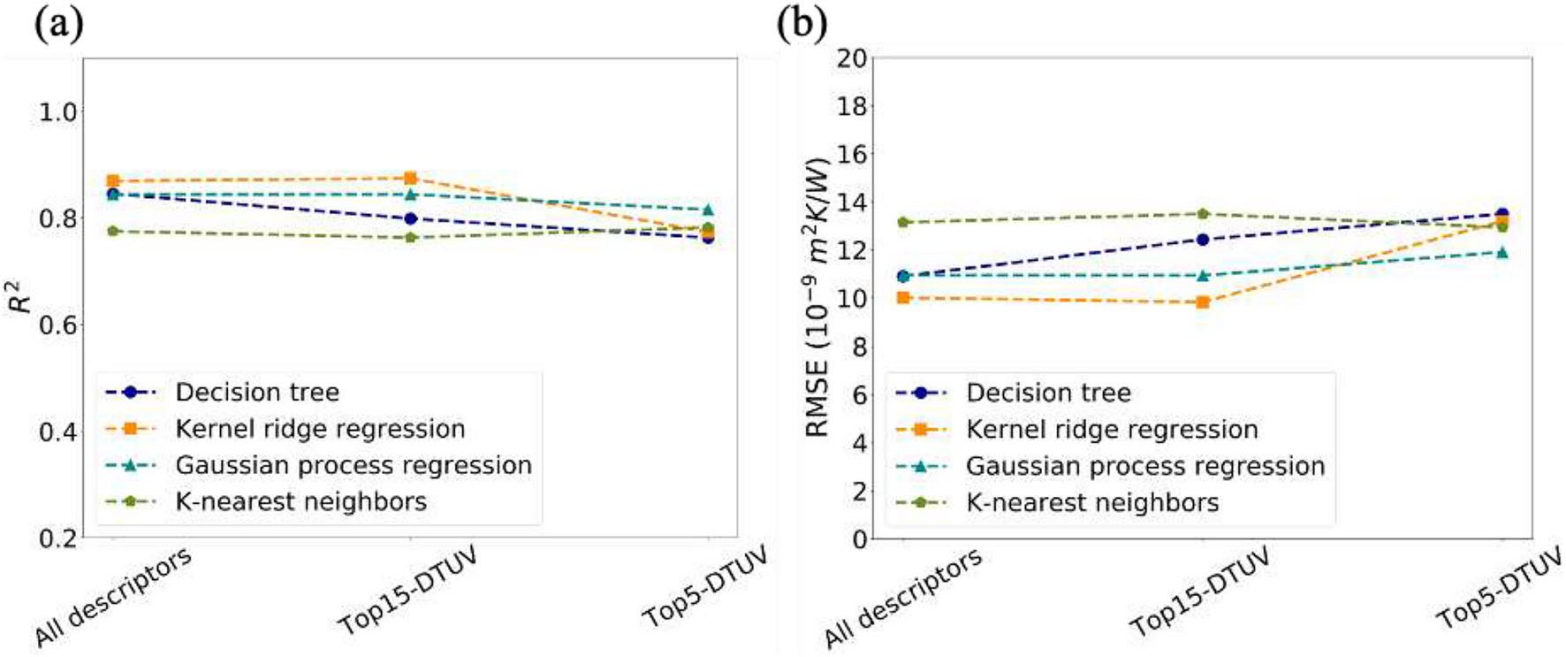
R^2^ (**a**) and RMSE (**b**) of test data (holdout set) predicted by models built by all descriptors, 15 common descriptors, and 5 common descriptors under shuffled cross-validation.

Performance of models built by the 15-common descriptors (Top15-DTUV) and 5-common (Top5-DTUV) descriptors were evaluated by R^2^ and RMSE under shuffled cross-validation. (Fig. 5) The same as the previous part, performance here is for the test data (holdout set). And train set performance is listed in Table S5 in supporting information. Unlike the descriptors selected by DT only, the descriptor reduction conducted by both DT and UV show a much more stable performance. The R^2^ of KRR improves from 0.62 to 0.77 by applying the top5-DTUV instead of top5-DT. And the R^2^ of GPR and R^2^ of KNN improves from 0.74 to 0.82 and from 0.65 to 0.78 by the same way. At the same time, the RMSE of KRR, GPR and KNN decreases with the utilization of top5-DTUV. Therefore, descriptors selected by a combination of decision tree and univariate testing are more reliable than that selected by only one algorithm.

### Model performance by grouped cross-validation

**Figure 6:**
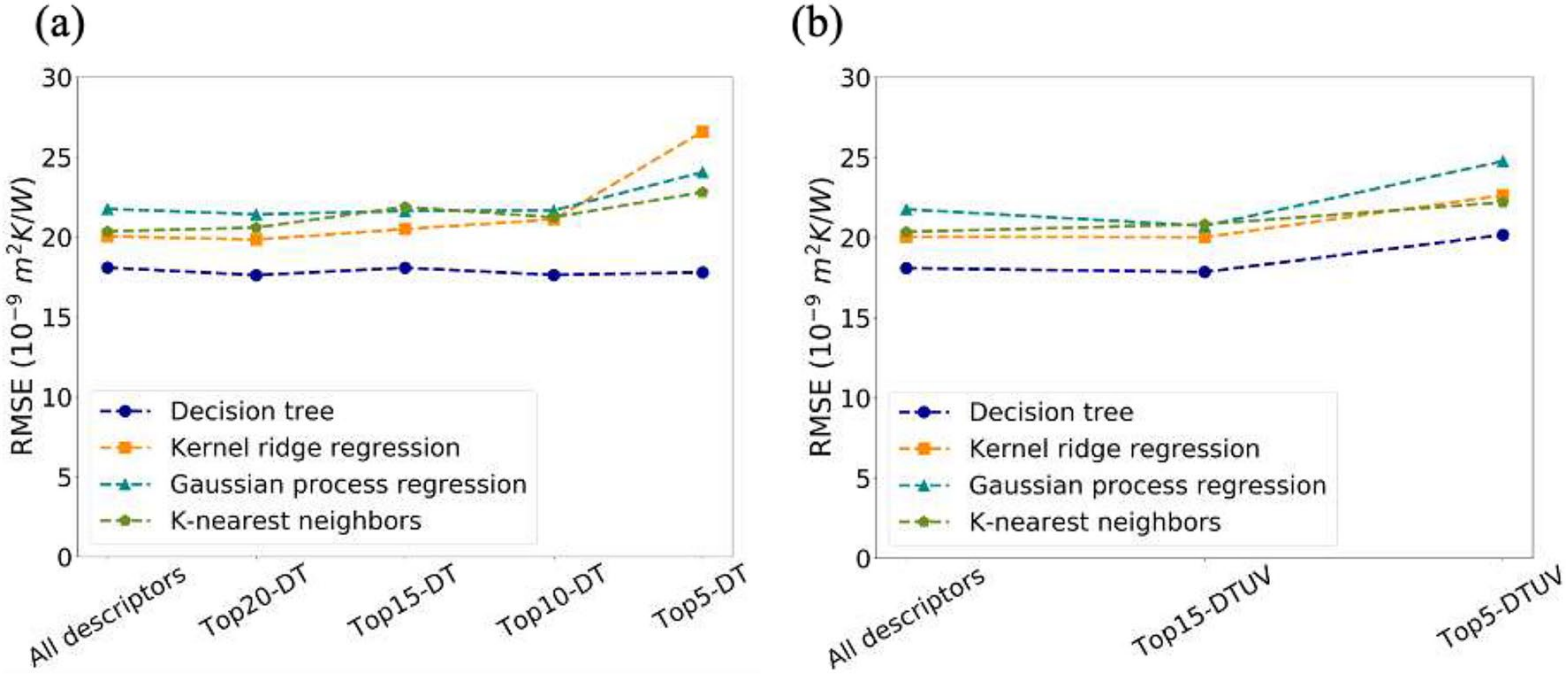
(**a**) RMSE of validation set by models built by descriptors selected by decision tree under grouped cross-validation. (**b**) RMSE of validation set by models built by common descriptors selected by decision tree and univariate testing under grouped cross-validation.

Beside shuffled cross-validation applied above, grouped cross-validation was investigated. The dataset was split by substrate/interlayer/film systems to guarantee that no identical interface exists in more than one group. Every group includes several distinct interface systems, ~ 20% of which serves as the validation set. In this case, it is not appropriate to have holdout set since no individual group can represent the others. Figure 6 shows the performance of models built by different descriptors set. The models RMSE for grouped cross-validation is not as good as that of random validation, which is not surprising since the information from many interfaces in validation set is not seen and learnt by machine learning models. Figure 6 shows RMSE values here are around 17 ~ 25, still lower than that from AMM and DMM models, which are 121 and 91^9^, respectively, confirming the superiority of our models built by grouped cross-validation.

As shown in Fig. 6, DT is best and the most robust among these models. For KNN and KRR, the descriptors selected by both DT and UV show much stable performance than descriptors selected by DT only, which is consistent with the conclusions drawn from shuffled cross-validation. In sum, shuffled cross-validation and grouped cross-validation were both performed in our work. In both systems, the common descriptors from decision tree and univariate testing are more reliable than that selected by only one algorithm. And the selection methods result in concise models with relatively good performance but much lower dimensions.

Although machine learning processes are hard to be understood intuitively, our findings (top5-DTUV) can be explained by classic theories and are well supported by experimental results documented in the literature. For example, melting point directly affect Lennard–Jones interatomic potential by the following equations in molecular simulations^66,67^:

$${T}_{m}\propto \varepsilon $$

$$4\varepsilon [{\left(\frac{\sigma }{r}\right)}^{12}- {\left(\frac{\sigma }{r}\right)}^{6}]$$

where ${T}_{m}$ is the melting point, $\varepsilon$ is the well depth, $\sigma$ is the distance at which the intermolecular potential equals to 0, $r$ is the real distance of both particles. Additionally, phonon transport is a dominating mode in thermal transport of nanostructured devices. Capacity (${C}_{V}$) is a key value when calculating phonon mean free path (${l}_{ph}$) based on kinetic theory^68^:

$${l}_{ph}= \frac{3{k}_{L}}{{C}_{V}{v}_{m}}$$

where ${v}_{m}$ is the average sound speed, ${k}_{L}$ is the lattice thermal conductivities. In other words, the effect of heat capacity on ITR is realized by affecting the phonon transport in nanostructured devices. The effect of electronegativity on ITR varies in different material systems. Thus, the analysis has to be on a case-by-case basis. Overall, electronegativity is always one of the most important factors in both theoretical and experimental exploration of ITR among various material systems^69–71^.

## Conclusions

In conclusion, descriptors selection for ITR prediction was conducted utilizing machine learning methods. Decision tree and univariate testing were applied to determine the important descriptors. Decision tree, kernel ridge regressor, Gaussian process regressor, and K-nearest neighbors were utilized to build models. Dataset was treated by shuffled cross-validation and grouped cross-validation. Performance of different algorithms and descriptors subsets were evaluated by R^2^ and RMSE. All models demonstrated relatively good performance when reducing all descriptors to top10-DT, indicating the validity of these selected descriptors. Furthermore, the 5 common descriptors selected both by top10-DT and top10-UV have a higher prediction accuracy than descriptors selected only by DT. These descriptors selected by machine learning methods based on big data collected from real experiments agree with properties affecting ITR heavily from a physical point of view. The characteristic selection methods by machine learning algorithms can not only be used for ITR prediction but also determining important descriptors for other materials properties.

## Supplementary Information

Supplementary Information
