# Supplementary material for: Descriptor selection for predicting interfacial thermal resistance by machine learning methods
Source: Sci Rep. 2021 Jan 12;11:739. doi: 10.1038/s41598-020-80795-z (PMC7804206; doi:10.1038/s41598-020-80795-z)
Supplement: Supplementary file 1 — Supplementary Information [file 41598_2020_80795_MOESM1_ESM.pdf]

Descriptor Selection for Predicting Interfacial Thermal Resistance by Machine Learning Methods

Xiaojuan Tian[1 *], Mingguang Chen[2*]

[1]State Key Laboratory of Heavy Oil Processing, China University of Petroleum, Beijing 102249, China.

[2]Physical Science and Engineering Division, King Abdullah University of Science and Technology (KAUST), Thuwal 23955-6900, Saudi Arabia.

Emails: tian@cup.edu.cn; mingguang.chen@kaust.edu.sa

* Corresponding authors' E-mails: tian@cup.edu.cn; mingguang.chen@kaust.edu.sa

Model	Grid search space	Hyperparameters picked
Decision tree	'max_depth':[5, 6, 7, 8, 9, 10, 11]<br>'min_samples_leaf':[1, 2, 3, 4]	'max_depth': 8,<br>'min_samples_leaf': 2
Kernel ridge regressor	'alpha': 10**(-2) to 10*11,<br>'gamma':10**(-9) to 10**3	'alpha': 0.01<br>'gamma': 0.1
Gaussian process regressor	'alpha':[0.05, 0.1, 0.5, 1],<br>'kernel':[RBF(0.001), RBF(0.005), RBF(0.01), RBF(0.05), RBF(0.1), RBF(0.5), RBF(1)]	'alpha': 0.5<br>'kernel': RBF(length_scale=0.5)
KNN	'n_neighbors':1-11,<br>'weights':['uniform', 'distance'],<br>'p':1-6	'n_neighbors': 3<br>'p': 1<br>'weights': 'distance'

Table S1. Grid search space and picked hyperparameters for various machine learning models trained by shuffled cross-validation.

Model	Grid search space	Hyperparameters picked
Decision tree	'max_depth':[2, 3, 5, 6, 7, 8, 9],<br>'min_samples_leaf':[1, 2, 3, 4, 5, 6, 7]	'max_depth':9,<br>'min_samples_leaf': 5
Kernel ridge regressor	'alpha': [0.0005, 0.001, 0.005, 0.01, 0.05, 0.1]<br>'gamma': [0.0001, 0.0005, 0.01, 0.05, 0.1, 0.5, 1]	'alpha': 0.05<br>'gamma': 0.01
Gaussian process regressor	'alpha':[0.05, 0.1, 0.5, 1, 5, 10]<br>'kernel':[RBF(0.001), RBF(0.005), RBF(0.01), RBF(0.05), RBF(0.1), RBF(0.5), RBF(1), RBF(10), RBF(100)]	'alpha': 1<br>'kernel': RBF(length_scale=0.5)

Table S2. Grid search space and final hyperparameters for various machine learning models trained by grouped cross-validation.

	DT				KRR				GPR				KNN			
	Train set		Test set		Train set		Test set		Train set		Test set		Train set		Test set	
	R^2	RMSE	R^2	RMSE	R^2	RMSE	R^2	RMSE	R^2	RMSE	R^2	RMSE	R^2	RMSE	R^2	RMSE
All descriptors	0.97	5.98	0.85	10.91	0.94	8.12	0.87	10.01	0.90	10.17	0.84	10.94	0.98	3.46	0.78	12.46
Top20-DT	0.97	5.98	0.85	10.91	0.93	8.26	0.87	9.90	0.91	9.93	0.85	10.68	0.98	3.60	0.78	13.00
Top15-DT	0.97	5.98	0.84	11.01	0.93	8.50	0.86	10.39	0.91	9.85	0.85	10.69	0.98	3.60	0.76	13.55
Top10-DT	0.96	6.65	0.88	9.78	0.91	9.76	0.85	10.57	0.90	10.15	0.83	11.25	0.98	4.35	0.73	14.44
Top5-DT	0.95	7.10	0.84	10.93	0.60	20.49	0.62	17.00	0.85	12.30	0.74	14.18	0.97	5.29	0.65	16.35

Table S3. R^2 and RMSE of models built by descriptor subsets selected by decision tree under shuffled cross-validation

	Descriptors
Top20-UV	T, fmass, fEb, smass, fthick, fmelt, fdensity, fAC1x, fAC1y, fAC2x, fAC2y, smelt, sAC1y, sIPc, fheatcap, funit, fENc, sheatcap, sunit, sENc
Top10-UV	fmass, fmelt, fAC1y, fAC2y, sIPc, fheatcap, funit, fENc, sheatcap, sENc

Table S4. Descriptor subsets selected by univariate testing

	DT				KRR				GPR				KNN			
	Train set		Test set		Train set		Test set		Train set		Test set		Train set		Test set	
	R^2	RMSE	R^2	RMSE	R^2	RMSE	R^2	RMSE	R^2	RMSE	R^2	RMSE	R^2	RMSE	R^2	RMSE
All descriptors	0.97	5.98	0.85	10.91	0.94	8.12	0.87	10.01	0.90	10.17	0.84	10.94	0.98	3.46	0.78	12.46
Top15-DTUV	0.96	6.05	0.80	12.43	0.93	8.73	0.87	9.83	0.90	10.17	0.84	10.94	0.98	4.35	0.76	13.44
Top5-DTUV	0.92	8.95	0.76	13.50	0.80	14.36	0.77	13.20	0.86	11.96	0.82	11.91	0.96	6.78	0.78	12.94

Table S5. R^2 and RMSE of models built by subsets selected by both decision tree and univariate testing under shuffled cross-validation
